# Attacking MALT1 for ABC-DLBCL therapy

**DOI:** 10.18632/oncotarget.794

**Published:** 2012-12-23

**Authors:** Daniel Krappmann

**Affiliations:** Helmholtz Zentrum München – German Research Center for Environmental Health, Research Unit Cellular Signal Integration, Institute of Molecular Toxicology and Pharmacology, Neuherberg, Germany

**Figure 1:**
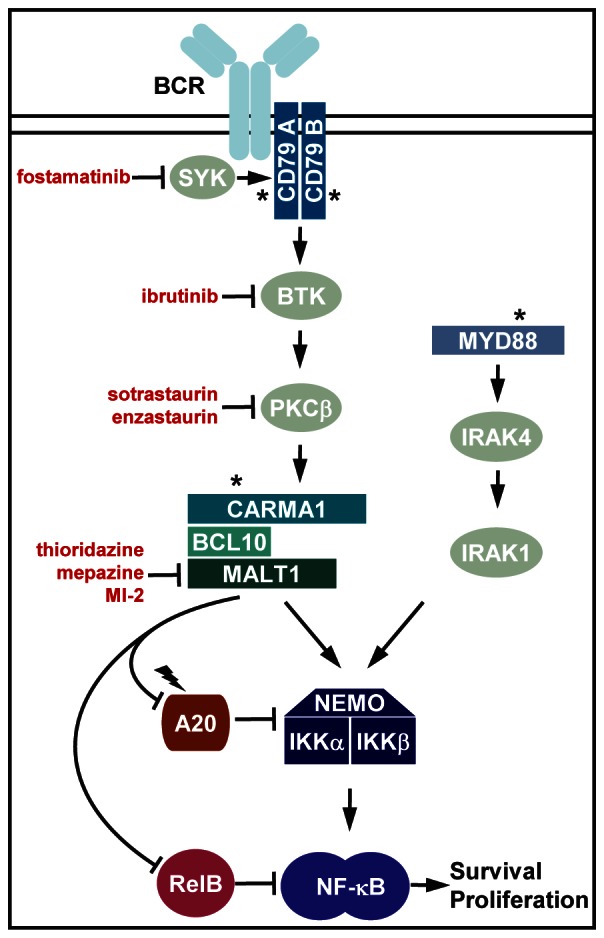
Oncogenic mechanisms and target directed therapeutic approaches in ABC-DLBCL Chronic BCR signaling engages Syk, CD79A/B, Btk and PKCβ to induce the recruitment of the CARMA1-BCL10-MALT1 (CBM) complex that triggers IKK/NF-κB activation. Activation of the MALT1 protease promotes lymphoma survival by cleaving and inactivating negative regulators of NF-κB, e.g. A20 and RelB. In a subset of ABC-DLBCL NF-κB is activated by constitutive signaling of the innate immune adaptor MYD88. Recurrent oncogenic (asterisk) or inactivating (flash) mutations are depicted. Drugs currently evaluated in preclinical and/or clinical studies and their molecular targets are indicated.

Diffuse large B-cell lymphoma (DLBCL) comprises the largest group of non-Hodgkin lymphoma (NHL). DLBCL can be classified according to their cellular origin and patients with the activated B-cell (ABC) subtype have an inferior prognosis relative to those with the germinal center B-cell (GCB) subtype. Constitutive anti-apoptotic NF-κB signaling is a hallmark of ABC-DLBCL. NF-κB activation is caused by chronic active B-cell receptor (BCR) signaling or constitutive MYD88 signaling. Oncogenic mutations that contribute to pathogenesis of ABC-DLBCL tumors have been identified in critical components of both pathways (Figure 1) [1].

Target directed approaches for ABC-DLBCL therapy have largely focused on the inhibition of upstream protein kinase [2]. Chronic BCR signaling engages the adaptors CD79A and CD79B in a Syk-dependent mechanism. Syk is constitutively active in many B-cell lymphomas and a clinical phase I/II trial using the Syk inhibitor fostamatinib disodium (FosD, AstraZeneca) shows some response also in DLBCL patients. However, most oncogenic mutations in ABC-DLBCL occur further downstream revealing that Syk may not be an optimal target. Downstream of CD79A/B, Btk and PKCβ bridge proximal BCR signaling events to the CARMA1 (CARD11)-BCL10-MALT1 (CBM) complex. Over 20% of ABC-DLBCL tumors carry oncogenic mutations in CD79A/B. Indeed, the irreversible Btk inhibitor ibrutinib (PCI-32765, Pharamcyclics) and the panPKC inhibitor sotrastaurin (STN) are inhibiting the outgrowth of CD79 mutant ABC-DLBCL in preclinical models [3, 4]. Furthermore, positive clinical responses in a phase II trial were reported in relapsed/refractory DLBCL with the selective PKCβ inhibitor enzastarin (LY317615, Eli Lilly) [5]. However, none of these potential drugs is able to target ABC-DLBCL tumors with lesions downstream of PKCβ or in parallel pathways, such as CARMA1 of MYD88, respectively.

Downstream of PKCβ the CBM subunit MALT1 has attracted great attention as a potential therapeutic target [6]. MALT1 serves a dual role in NF-κB signaling in response to antigen receptor stimulation. MALT1 is an adaptor that recruits and activates the IκB kinase (IKK) complex, the gatekeeper of canonical NF-κB. In addition, MALT1 is a paracaspase with a caspase-like proteolytic activity that is required for full NF-κB signaling and survival of ABC-DLBCL cells. MALT1 cleaves and thereby inactivates negative regulators of canonical NF-κB, such as the tumor suppressor A20 and the NF-κB subunit RelB which both counteract pro-survival functions of canonical IKK/NF-κB signaling.

Two classes of MALT1 inhibitors have now been identified that effectively and selectively inhibit the growth of ABC- but not GCB-DLBCL in vitro and in vivo [7, 8]. Interestingly, the compounds are inhibiting MALT1 by two very different mechanisms. Fontan et al have identified a structurally new small molecule inhibitor (MI-2) that is covalently modifying catalytic center of MALT1 [7]. MI-2 was tolerated in mice at the effective dose without obvious signs of toxicity. Usually, irreversible inhibitors require optimal pharmacokinetic properties for clinical development, but recent advances for instance on the irreversible Btk inhibitor ibrutinib reveal that a clinical use may be possible. In a parallel study, we have identified the phenothiazines-derivatives (PD) thioridazine, mepazine and promazine as reversible MALT1 inhibitors [8]. PD are not targeting the active site of MALT1, but exhibit a non-competitive, allosteric mode of action. Mepazine, thioridazine and promazine have a long medical history as antipsychotics and sedatives drugs used for the treatment of psychiatric disorders. Well-established toxicokinetics and pharmacokinetics suggest that targeting MALT1 for cancer therapy by this class of compounds may be safe and feasible. Further, medicinal chemistry could be used to generate novel PD that are more potent MALT1 inhibitors, while reducing their neurological effects. Taken together, both studies demonstrate that MALT1 inhibition is a promising strategy for the treatment of ABC-DLBCL. In fact, targeting MALT1 may possess some advantages over the inhibition of upstream protein kinases. MALT1 inhibition also affects survival of CARMA1 mutant ABC-DLBCL. Further, with an occurrence of 29% the MYD88 mutation L265P is the most frequent oncogenic mutation in ABC-DLBCL. 65% of the MYD88-mutant ABC-DLBCL tumors carry additional mutations in CARMA1 or CD79A/B and MALT1 inhibitors are toxic to ABC-DLBCL with aberrant activation of both pathways [7, 8]. Thus, MALT1 inhibition indeed holds great promises for the treatment of the majority of ABC-DLBCL.

Research on ABC-DLBCL provides a paradigm for the power of using advanced diagnostic tools (e.g. gene expression profiling and genome sequencing) to classify lymphoma entities and to identify specific oncogenic lesions. In parallel the generation of target directed therapeutics will promote the development of more personalized treatment protocols. Because of the various oncogenic lesions and the possibilities of drug resistances, there cannot be enough ‘smart’ drugs for ABC-DLBCL therapy. In the future, combinatorial treatment protocols may certainly enhance anti-tumor efficacy of the single agents that are currently in preclinical and/or clinical development.
